# First report of multiple infection by *Anaplasma platys*, *Bartonella henselae*, and hemoplasmas in clinically healthy domestic cats from Southern Brazil

**DOI:** 10.1007/s11259-026-11107-5

**Published:** 2026-03-14

**Authors:** Paola Renata Joanol Dallmann, Diago Dutra Lima, Camila Xavier Silveira, Victória da Rosa Leite Silva, Pedro Machado Medeiros de Alburquerque, Luiz Filipe Damé Schuch, Rodrigo Casquero Cunha

**Affiliations:** Department of Preventive Veterinary Medicine, Faculty of Veterinary Medicine, Federal University of Pelotas, University Campus, RS S/N-CEP 96160-000 Capão do Leão, Brazil

**Keywords:** Cats, Diagnosis, PCR, Coinfection, Zoonoses

## Abstract

Hemoparasitoses in domestic cats are increasingly recognized as an emerging challenge for veterinary medicine and public health due to their subclinical persistence and zoonotic potential. This study aimed to detect *Anaplasma platys*, *Bartonella henselae*, and hemotropic mycoplasmas (*Mycoplasma haemofelis* [*Mhf*] and ‘*Candidatus* Mycoplasma haemominutum’ [*C*Mhm]) in clinically healthy cats from Pelotas, southern Brazil, using molecular assays. A total of 151 blood samples collected between 2022 and 2024 were analyzed by conventional and nested PCR. Detection rates were 21.9% (33/151) for *(A) platys*, 15.2% (23/151) for *(B) henselae*, and 21.9% (33/151) for hemoplasmas, with a higher occurrence of *C*Mhm (17.2%; 26/151) than *Mhf* (4.0%; 6/151). Coinfections occurred in 10.7% (16/151) of cats, including dual infections and one triple infection (0.7%; 1/151) involving *(A) platys*, *(B) henselae*, and *C*Mhm. Statistical analyses showed that *(A) platys* infection was significantly associated with adult age (*p* = 0.01), *(B) henselae* was more frequent in kittens (*p* = 0.04), and hemoplasma infection occurred more often in males (*p* = 0.03). To our knowledge, this is the first molecular evidence of concurrent infection *by (A) platys*, *(B) henselae*, and ‘*Candidatus* Mycoplasma haemominutum’ in a cat from Brazil. These findings highlight the influence of host factors on pathogen dynamics and reinforce the need for molecular surveillance and integrated vector control under a One Health perspective.

## Introduction

Hemoparasitoses in domestic cats represent a growing challenge for veterinary medicine, not only because of their clinical impact on affected animals but also due to the zoonotic potential of several agents involved. Among them, hemotropic mycoplasmas — *Mycoplasma haemofelis* (*Mhf*) and ‘*Candidatus* Mycoplasma haemominutum’ (*C*Mhm) — can cause hemolytic anemia of variable severity, although they frequently persist as subclinical infections (Sykes 2010; Tasker 2010; Dallmann et al. 2025).

Another emerging agent is *Anaplasma platys*, traditionally associated with infectious cyclic thrombocytopenia in dogs. In cats, infection is often subclinical or associated with subtle hematological changes, making clinical diagnosis difficult (André et al. 2022; Almeida et al. 2022). In parallel, *Bartonella henselae*, the causative agent of cat scratch disease, finds its main reservoir in felines, maintaining chronic bacteremia that is usually asymptomatic and therefore represents a significant risk to human health (Staggemeier et al. 2010; Bush et al. 2024).

The interaction between multiple agents may alter the host’s immune response, hinder clinical diagnosis, and increase the risk of silent dissemination. In this context, detecting these pathogens in asymptomatic cats is essential to understanding local epidemiology and supporting preventive strategies within a One Health framework. The present study aimed to detect *(A) platys*, *(B) henselae*, and hemoplasmas in clinically healthy domestic cats using polymerase chain reaction (PCR) assays.

## Materials and methods

### Sampling

A total of 151 blood samples from clinically healthy domestic cats were collected between 2022 and 2024 at a veterinary clinic in Pelotas, Rio Grande do Sul, Brazil. All animals showed no alterations upon physical examination and were presented for routine checkups or elective surgical procedures. Cats were included regardless of age, sex, or breed. All animals tested negative for Feline Immunodeficiency Virus (FIV) and Feline Leukemia Virus (FeLV). Sample selection, DNA extraction, and hemoplasma detection were conducted as previously described (Dallmann et al. 2025). All sample processing and molecular analyses were carried out at the Laboratório de Biologia Molecular Veterinária (LaBMol-Vet), Universidade Federal de Pelotas (UFPel).

## Extraction and quantification of DNA

Genomic DNA (gDNA) was extracted from the samples using a chemical extraction method with a phenol-guanidine-based reagent (Brazol™). The purity and quantity of the extracted material were assessed by ultraviolet spectrophotometry using a NanoDrop^®^ spectrophotometer (Thermo Fisher Scientific, Waltham, MA, USA). Samples showing an A260/A280 ratio between 1.8 and 2.0 were considered suitable for PCR assays. After quantification, DNA samples were stored at -80 °C until PCR assays were performed.

## Molecular detection by PCR

Molecular detection of the agents was carried out using conventional and nested PCR. All amplifications were carried out in a final reaction volume of 25 µL, containing 2.5 µL of 10× PCR buffer, 0.5 µL of dNTPs (0.2 mM each), 1.25 µL of MgCl₂ (1.5 mM), 0.5 µL of each primer (0.2 pmol), 0.25 µL (1.25 U) of Taq DNA polymerase (Ludwig Biotecnologia^®^, Alvorada, RS, Brazil), 16.5 µL of ultrapure water (Ludwig Biotecnologia^®^, Alvorada, RS, Brazil), and 2 µL of genomic DNA.

Detection of *(A) platys* was performed by conventional PCR targeting the 16 S rRNA gene using the primers PLATYS and EHR16SR, generating an expected amplicon of approximately 678 bp. The amplification conditions consisted of an initial denaturation at 94 °C for 2 min, followed by 40 cycles of denaturation at 94 °C for 30 s, annealing at 55 °C for 30 s, and extension at 72 °C for 30 s, with a final extension step at 72 °C for 5 min. These conditions were based on the protocol described by Martin et al. (2005), with minor modifications. Hemotropic mycoplasmas (*Mycoplasma haemofelis* and ‘*Candidatus* Mycoplasma haemominutum’) were detected by conventional PCR targeting the 16 S rRNA gene, using primers Hf-F and Hf-R, producing amplicons of 170 bp and 193 bp, respectively, following the protocol described by Jensen et al. (2001). Detection of *(B) henselae* was conducted by nested PCR targeting the *gltA* gene. The first amplification round was performed according to Rolain et al. (2003), generating fragments ranging from 700 to 722 bp, followed by a second amplification round as described by Sato et al. (2017), yielding a 254 bp fragment.

Quality assurance was maintained throughout all molecular procedures by including appropriate positive and negative controls in each PCR run. Positive controls consisted of DNA extracted from domestic cats previously confirmed as positive for the respective agents and belonging to the molecular collection of the Laboratório de Biologia Molecular Veterinária (LaBMol-Vet), Universidade Federal de Pelotas (UFPel). Negative controls consisted of ultrapure water and were included in all PCR reactions.

## Agarose gel electrophoresis

Amplified products were subjected to electrophoresis on 1.5% agarose gels stained with ethidium bromide and visualized under ultraviolet light using a UV transilluminator (Hoefer^®^, USA). Fragment sizes were estimated using a 100 bp DNA Ladder (Ludwig Biotecnologia^®^, Brazil).

### Statistical analysis

Associations between molecular positivity and epidemiological variables — including age, sex, outdoor access, presence of ectoparasites, and cohabitation with other animals — were evaluated for statistical significance using the Chi-square or Fisher’s exact test when appropriate. Variables were considered statistically significant at *p* ≤ 0.05. Odds ratios (OR) and their corresponding 95% confidence intervals (95% CI) were calculated to estimate the strength of association between infection status and each factor. For analytical purposes, cats testing positive for ‘*Candidatus* Mycoplasma haemominutum’, *Mycoplasma haemofelis*, or both were grouped as “hemoplasma-positive.” All analyses were performed using R statistical software (version 4.3.2; R Foundation for Statistical Computing, Vienna, Austria).

## Results

Molecular analysis of the 151 blood samples from clinically healthy cats revealed the presence of various hemoparasites. *Bartonella henselae* was detected in 15.2% (23/151) of the animals, while *A. platys* was present in 21.9% (33/151). Among hemoplasmas, also identified in 33 cats (21.9%), *C*Mhm was the most frequent, detected in 17.2% (26/151) of the animals, followed by *Mhf* in 4.0% (6/151). Only one animal (0.7%; 1/151) presented co-infection with *C*Mhm and *Mhf*.

Co-infections were notable in the analyzed cohort, highlighting cats simultaneously infected with different hemoparasites. Co-infections involving *C*Mhm and *B. henselae* were observed in 4.0% (6/151) of the cats, the same frequency of 4.0% (6/151) was recorded for the association between *B. henselae* and *A. platys*. Additionally, 2.0% (3/151) of the animals showed concurrent infection with *(A) platys* and *C*Mhm. Notably, a single case (0.7%; 1/151) of triple infection was detected, involving *(B) henselae*, *A. platys*, and *C*Mhm, underscoring the complexity of parasitic interactions in these hosts.

To explore potential risk factors associated with pathogen occurrence, statistical analyses were conducted considering host- and environment-related variables. Table 1 summarizes these associations in clinically healthy domestic cats. Adult cats were significantly more likely to be infected with *(A) platys* (*p* = 0.010), whereas *(B) henselae* infection was more frequent among kittens (*p* = 0.041). Male cats showed a higher likelihood of hemoplasma infection (*p* = 0.030). Although *A. platys* detection tended to be higher in cats with outdoor access (*p* = 0.052), this difference did not reach statistical significance. No significant associations were found between infection status and the presence of ectoparasites or environmental cohabitation.

**Table 1:** Risk factors associated with infection by *Anaplasma platys*, *Bartonella henselae*, and Hemoplasmas in clinically healthy domestic cats from Pelotas, RS, Brazil

Variables (*n*)	A. platys detection *n* (%)	OR (95% CI)	*P* value^1^	B. henselae detection *n* (%)	OR (95% CI)	*P* value^1^	Hemoplasmas detection *n* (%)	OR (95% CI)	*P* value^1^
Age									
Kitten (28)	1 (0.7)			8 (5.3)			8 (5.3)		
Adult (123)	32 (21.2)	0.11 (0.01–0.81)	**0.010**	15 (9.9)	2.88 (1.08–7.69)	**0.041**	25 (16.6)	1.57 (0.62–3.97)	0.325
Sex									
Male (74)	14 (9.3)			10 (6.6)			22 (14.6)		
Female (77)	19 (12.6)	0.71 (0.33–1.55)	0.435	13 (8.6)	0.77 (0.31–1.88)	0.653	11 (7.3)	2.54 (1.13–5.71)	**0.030**
Ectoparasite presence									
Yes (26)	8 (5.3)			5 (3.3)			7 (4.6)		
No (125)	25 (16.6)	1.78 (0.69–4.56)	0.295	18 (11.9)	1.42 (0.47–4.23)	0.552	26 (17.3)	1.40 (0.53–3.69)	0.602
Street access									
Yes (43)	14 (9.3)			6 (4.0)			11 (7.3)		
No (108)	19 (12.6)	2.26 (1.01–5.07)	0.052	17 (11.2)	0.87 (0.32–2.37)	1.000	22 (14.6)	1.34 (0.59–3.08)	0.516
Shared environment									
Yes (90)	19 (12.6)			13 (8.6)			21 (13.9)		
No (61)	14 (9.3)	0.90 (0.41–1.96)	0.842	10 (6.6)	0.86 (0.35–2.11)	0.819	12 (8.0)	1.24 (0.56–2.76)	0.690

OR = odds ratio; CI = confidence interval. ¹Chi-square test. Statistically significant values (*p* ≤ 0.05) are shown in bold

## Discussion

The detection of hemoparasites in clinically healthy cats indicates that these animals may act as asymptomatic reservoirs, maintaining the silent circulation of agents with veterinary and zoonotic importance. The presence of *B. henselae*, *A. platys*, and hemoplasmas, both alone and in co-infections, demonstrates a complex pattern of pathogen coexistence similar to that reported in other regions (Zhang et al. 2021; Almeida et al. 2022; Bush et al. 2024).

In Brazil, hemoparasites have also been reported in domestic cats without evident clinical signs, although involving different agents and diagnostic approaches, such as blood smear–based detection of *Babesia* spp., *Ehrlichia* spp. and *Mycoplasma* spp. (Pereira et al. 2025). In this context, the present study advances national knowledge by providing molecular evidence of the circulation and coinfection of vector-borne agents in clinically healthy cats using PCR-based methods.

Statistical analyses indicated that *(A) platys* infection was significantly associated with adult age, suggesting that cumulative exposure to arthropod vectors over time may contribute to infection persistence. In contrast, *(B) henselae* was more frequently detected in kittens, possibly reflecting early-life exposure followed by transient bacteremia (André et al. 2022; Almeida et al. 2022).

The higher occurrence of hemoplasma infection among males supports previous evidence that territorial behavior and increased aggression enhance susceptibility to vector-borne and blood-transmitted pathogens (Tasker 2010; Zhang et al. 2021). Although *A. platys* positivity tended to be higher in cats with outdoor access, this trend did not reach statistical significance, likely due to limited data on tick exposure.

Overall, these findings reinforce that clinically healthy cats may serve as asymptomatic reservoirs for multiple hemoparasites and that host-related factors—particularly age and sex—play a relevant role in shaping infection dynamics. Continuous molecular surveillance and rigorous vector control remain essential to mitigate transmission risks within urban and peri-urban environments.

The relatively high frequency of *A. platys* (21.9%; 33/151) observed here is notable, as this species is traditionally associated with dogs. Although its clinical significance in cats remains uncertain, our findings suggest that felines may have a more active role in the epidemiology of this agent than previously assumed (Zobba et al. 2015; Coelho et al. 2023). Further investigations, including experimental studies, are needed to clarify this potential relationship.

From a One Health perspective, the coexistence of *B. henselae* and *A. platys* in asymptomatic cats reinforces the importance of continuous molecular surveillance and ectoparasite control, since these infections may remain undetected and contribute to human exposure, especially in urban environments (Maggi et al. 2013; Day 2016).

Some inherent limitations of this study should be considered when interpreting the findings. The number and origin of samples, collected from a single locality, may limit the extrapolation of the results to other feline populations. In addition, detailed hematological analyses and vector investigations were not included, which could have further contributed to understanding the infection and transmission dynamics. Nevertheless, the results provide consistent evidence of the presence and coexistence of multiple hemoparasites in clinically healthy cats, reinforcing the importance of continuous molecular surveillance and integrated approaches in animal and public health.

## Conclusion

This study provides the first molecular evidence of concurrent infection by *(A) platys*, *(B) henselae*, and hemoplasmas in clinically healthy cats from southern Brazil. The observed association of *A. platys* with adult age and the higher frequency of hemoplasma infection among males underscore the influence of host factors on pathogen dynamics. These findings highlight the need for continuous molecular surveillance and integrated vector control strategies to clarify the epidemiological role of cats as reservoirs of vector-borne pathogens within a One Health framework.

## Data Availability

No datasets were generated or analysed during the current study.
